# Burden of unique and low prevalence somatic mutations correlates with cancer survival

**DOI:** 10.1038/s41598-019-41015-5

**Published:** 2019-03-19

**Authors:** Nikolai Klebanov, Mykyta Artomov, William B. Goggins, Emma Daly, Mark J. Daly, Hensin Tsao

**Affiliations:** 1 Wellman Center for Photomedicine, Massachusetts General Hospital, Harvard Medical School, Boston, MA USA; 2 Analytic and Translational Genetic Unit, Massachusetts General Hospital, Boston, MA USA; 3 Broad Institute, Cambridge, MA USA; 4 School of Public Health and Primary Care, The Chinese University of Hong Kong, Hong Kong, Hong Kong

## Abstract

Tumor mutational burden correlates with improved survival and immunotherapy response in some malignancies, and with tumor aggressiveness in others. To study the link between mutational burden and survival, we analyzed survival effects of tumor exonic missense mutation burden (TEMMB) across 6947 specimens spanning 31 cancers which have undergone whole exome sequencing as part of TCGA. We adjusted TEMMB for age, sex, stage, and recruitment center, and computed Cox-proportional models of TEMMB survival effects. We assigned a recurrence score (RS) to each cohort, defining RS as the burden of recurrent mutations exceeding 1% population prevalence. High TEMMB was associated with improved survival in cutaneous melanoma: hazard ratio (HR) = 0.71 [0.60–0.85], p = 0.0002, urothelial bladder carcinoma: HR = 0.74 [0.59–0.93], p = 0.01, and ovarian carcinoma: HR = 0.80 [0.70–0.93], p = 0.003. High TEMMB was associated with decreased survival in colorectal adenocarcinoma: HR = 1.32 [1.00–1.74], p < 0.05. We identified that TEMMB survival effects were governed by the balance of recurrent and non-recurrent mutations. In cancers with a low RS, high TEMMB was correlated with better survival outcomes (r = 0.49, p = 0.02). In conclusion, TEMMB effects on survival depend on recurrent mutation enrichment; tumor types that are highly enriched in passenger mutations show a survival benefit in the setting of high tumor mutational burden.

## Introduction

Tumor mutational burden has been described as a predictor of tumor behavior and immunological response^1–3^. At its core, mutation formation promotes carcinogenesis via activation or inactivation of genes and associated pathways, thus generating novel peptide sequences which can stimulate immune response. High mutational burden may in some cases represent a high underlying number of drivers, and indicate a higher-risk tumor: for example patients with high mutational burden lung adenocarcinoma tumors showed a 14-month survival decrease^4^, supporting that high mutation burden may be a harbinger of poor clinical outcomes. Alternatively, highly mutated tumors may develop many novel peptides and thus display more neoantigens, rendering them more susceptible T-cell targets^5^. For example, patients with melanomas with a high mutational load showed improved survival with ipilimumab^6^ and improved overall survival^7^; patients with highly mutated ovarian cancer had improved postoperative chemotherapy response and higher overall survival^2^.

Here, we systematically analyzed mutational burden survival effects across multiple cancer types. We hypothesized that tumor exonic missense mutational burden (TEMMB) is predictive of underlying total exonic mutational burden (TEMB), and that TEMMB is independent of critical demographic and tumor-specific factors. Furthermore, we hypothesized that TEMMB is a predictive marker of tumor immune surveillance and clinical outcomes. We sought to test these hypotheses, and to describe the potential genetic underpinnings for the impact of TEMMB on survival. We focused on somatic missense mutation burden in subsequent analyses. Missense mutations represent the most common observed oncogenic variants^8^, and are known to alter sequences of expressed transcripts and thus lead to downstream translation of mutated proteins^9^. Furthermore, missense variants specifically have been suggested to be the most frequent class of alterations to carry the potential for neoepitope generation in chronic lymphocytic leukemia malignancy (as compared to frameshift or splice-site variants)^10^. In multiple myeloma, missense mutational load was found to be highly correlated with predicted neoantigen loads^11^. Missense mutations produce specific amino acid changes in a known pattern, allowing for a systematic way to characterize mutational profiles by defining recurrent and non-recurrent mutations.

## Results

### Tumor missense mutational burden (TEMMB) variability among cancers

Total missense mutational burden across all cohorts ranged from a low of 8 (median) missense mutations among acute myeloid leukemia (LAML) and thymoma (THYM), to 256 median mutations among the skin cutaneous melanoma (SKCM) cohort (Fig. S1). 10 individuals were removed as TEMMB outliers (Fig. S2). Total (TEMB) and missense (TEMMB) tumor exonic mutational burden were found to be closely correlated among all cohorts: Pearson’s r ranged from 0.95–1.00 for all cohorts other than uveal melanoma (UVM) which also revealed a strong positive correlation with r = 0.88 likely due to a small (N = 79) sample size (p < 2.2 × 10^−16^ for all cohorts) (Fig. S3).

### TEMMB relations to age, sex and tumor stage

Increasing patient age was significantly correlated with high TEMMB among 17 of 31 (55%) cohorts (Table 1). Male sex was significantly associated with high TEMMB in renal papillary cell carcinoma (KIRP), sarcoma (SARC), and cutaneous melanoma (SKCM). Female sex was significantly associated with high TEMMB in colorectal adenocarcinoma (COAD) and glioblastoma multiforme (GBM). High tumor stage (Stage III and above) was observed to be significantly associated with both high TEMMB in 3 cohorts and low TEMMB in 7 cohorts.

**Table 1:** Contributions of age, sex, and tumor stage to tumor exonic missense mutational burden (TEMMB). Each model was additionally adjusted by recruitment center (IRR and p-values not shown).

	Age		Sex			Tumor Stage		
	*Age*, *yrs (IRR*^a^)	*p* ^b^	*Fem (ref*)	*Male (IRR*^a^)	*p* ^b^	*I-II (ref)*	*III* + *(IRR*^a^*)*	*p* ^b^
ACC	1.02 [1.01–1.03]	<0.05/31**	1	0.78 [0.57–1.09]	0.14	1	1.35 [0.99–1.85]	0.06
BLCA	1.00 [0.99–1.02]	0.56	1	1.30 [0.95–1.76]	0.08	1	1.37 [1.03–1.80]	<0.05*
BRCA	1.01 [1.01–1.02]	<0.05/31**	1	0.64 [0.35–1.33]	0.19	1	0.78 [0.67–0.91]	<0.05/24**
CESC	1.02 [1.01–1.03]	<0.05/31**	1	—	—	1	1.09 [0.76–1.61]	0.63
CHOL	1.01 [1.00–1.01]	0.07	1	0.93 [0.80–1.08]	0.32	1	0.81 [0.68–0.97]	<0.05*
COAD	1.00 [0.98–1.01]	0.76	1	0.57 [0.39–0.83]	<0.05*	1	0.50 [0.34–0.74]	<0.05/24**
DLBC	1.00 [0.99–1.02]	0.70	1	1.26 [0.80–1.97]	0.31	1	0.57 [0.33–1.01]	<0.05*
ESCA	1.01 [1.00–1.02]	<0.05*	1	0.89 [0.71–1.11]	0.32	1	0.99 [0.83–1.19]	0.95
GBM	1.01 [1.00–1.01]	<0.05/31**	1	0.87 [0.80–0.94]	<0.05/26**	1	—	—
HNSC	1.01 [1.01–1.02]	<0.05/31**	1	1.04 [0.85–1.27]	0.69	1	1.00 [0.80–1.23]	0.98
KICH	1.00 [1.00–1.01]	0.09	1	0.93 [0.79–1.09]	0.37	1	1.25 [1.06–1.47]	<0.05*
KIRC	1.01 [1.01–1.02]	<0.05/31**	1	1.04 [0.91–1.18]	0.57	1	1.01 [0.89–1.16]	0.87
KIRP	1.01 [1.01–1.02]	<0.05/31**	1	1.20 [1.07–1.34]	<0.05/26**	1	1.11 [1.00–1.25]	0.06
LAML	1.01 [1.00–1.02]	<0.05/31**	1	1.11 [0.91–1.36]	0.30	1	—	—
LGG	1.02 [1.02–1.03]	<0.05/31**	1	0.94 [0.85–1.04]	0.21	1	—	—
LIHC	1.00 [1.00–1.01]	0.24	1	1.06 [0.86–1.29]	0.58	1	0.94 [0.76–1.17]	0.59
LUAD	1.00 [0.99–1.01]	0.99	1	1.18 [0.92–1.50]	0.19	1	0.81 [0.62–1.07]	0.13
LUSC	0.99 [0.98–1.01]	0.35	1	1.04 [0.83–1.28]	0.75	1	1.01 [0.80–1.29]	0.91
OV	1.01 [1.00–1.01]	<0.05/31**	1	—	—	1	0.67 [0.50–0.89]	<0.05*
PAAD	1.00 [0.99–1.01]	0.92	1	1.11 [0.97–1.27]	0.14	1	1.11 [0.82–1.53]	0.51
PCPG	1.01 [1.01–1.02]	<0.05/31**	1	1.06 [0.93–1.22]	0.38	1	—	—
PRAD	1.00 [0.99–1.01]	0.84	1	—	—	1	—	—
READ	0.98 [0.96–1.00]	0.05	1	0.92 [0.61–1.38]	0.68	1	0.56 [0.37–0.85]	<0.05*
SARC	1.02 [1.01–1.03]	<0.05/31**	1	1.43 [1.17–1.75]	<0.05/26**	1	—	—
SKCM	1.01 [1.00–1.02]	<0.05*	1	1.39 [1.11–1.72]	<0.05*	1	0.93 [0.74–1.17]	0.51
STAD	1.02 [1.00–1.03]	<0.05*	1	0.95 [0.68–1.32]	0.72	1	0.95 [0.69–1.30]	0.72
THCA	1.01 [1.01–1.02]	<0.05/31**	1	0.96 [0.85–1.08]	0.49	1	1.14 [1.00–1.30]	<0.05*
THYM	1.03 [1.02–1.05]	<0.05/31**	1	1.19 [0.85–1.66]	0.31	1	—	—
UCEC	0.98 [0.96–0.99]	0.14	1	—	—	1	0.79 [0.47–1.37]	0.31
UCS	1.00 [0.98–1.02]	0.89	1	—	—	1	1.20 [0.88–1.63]	0.21
UVM	1.00 [1.00–1.01]	0.43	1	1.13 [0.96–1.34]	0.14	1	0.81 [0.67–0.98]	<0.05**

Older age correlated with high TEMMB in 17 of 31 cancers studied. Effects of male sex and high tumor stage (defined as Stage III or greater) were variable.

^a^IRR: Incidence rate ratio calculated with multivariate binomial regression, reported with 95% confidence intervals.

^b^p-values displayed with conventional 0.05 significance cutoff and with cutoff using Bonferroni correction for multiple comparisons (n = 31, 26, 24 for age, sex, stage respectively).

### Melanoma, ovarian carcinoma, and bladder carcinoma benefit from high mutational load

**Figure 1:**
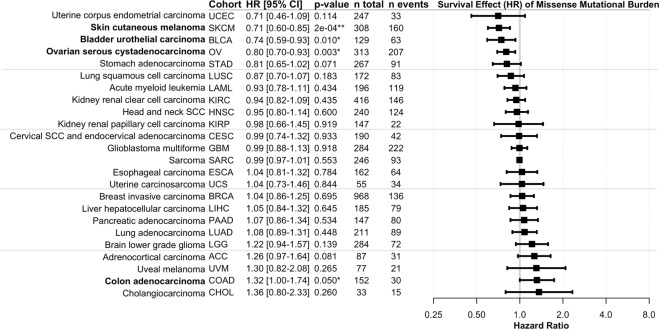
Survival effects of tumor exonic missense mutational burden (TEMMB). Survival is expressed as hazard ratio (HR) per effective multivariate-adjusted TEMMB. Raw two-sided Wald-test p-values are reported with *indicating p < 0.05 and **indicating Bonferroni-adjusted significance for 24 multiple comparisons. Ovarian carcinoma, cutaneous melanoma, bladder carcinoma, and colorectal adenocarcinoma showed significant survival benefit with high TEMMB.

Following multivariate adjustment for age, sex, stage, and patient recruitment center and exclusion of seven cohorts with a low number of non-censored events, TEMMB was found to be significantly correlated with survival in 4 of 24 TCGA cohorts (Fig. 1). High TEMMB correlated with improved survival in skin cutaneous melanoma (SKCM): HR = 0.71 [0.60–0.85], p = 0.0002, bladder urothelial carcinoma (BLCA): HR = 0.74 [0.59–0.93], p = 0.01, and ovarian carcinoma (OV): HR = 0.80 [0.70–0.93], p = 0.003. High TEMMB was associated with decreased survival in colorectal adenocarcinoma (COAD): HR = 1.32 [1.00–1.74], p < 0.05 (p = 0.0497). Following Bonferroni adjustment of α = 0.05 for 24 comparisons, yielding a p-value cutoff of α/24 = 0.0021, only cutaneous melanoma retained a significant correlation between TEMMB and survival.

### Relative burden of recurrent and non-recurrent mutations expressed with recurrence score (RS)

**Figure 2:**
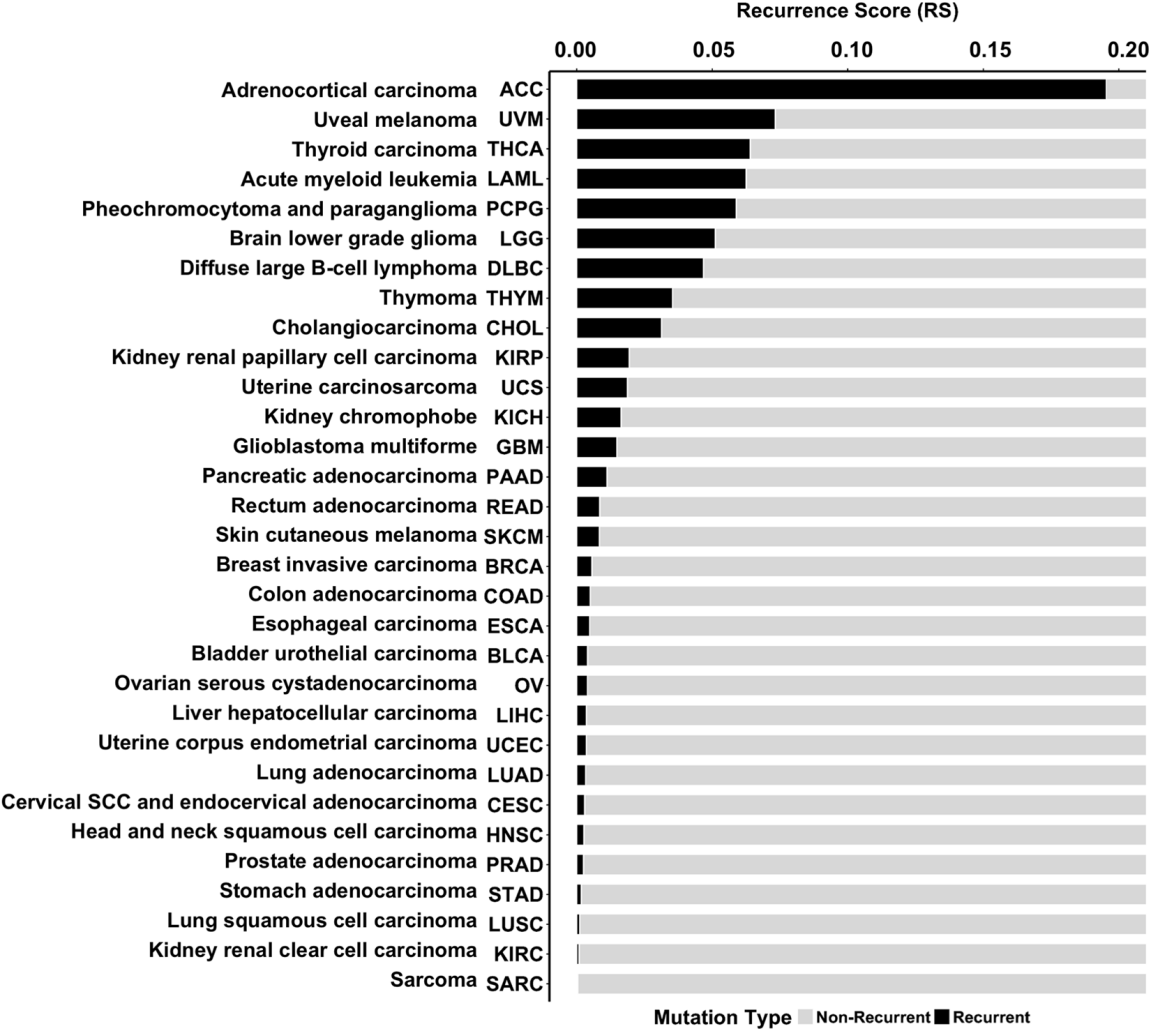
Recurrence scores (RS) of all cancer cohorts, calculated as the fraction of recurrent missense mutations to total missense mutations in the pool. Recurrent mutations were defined as those which exceeded 1% prevalence in the cohort.

To characterize the somatic mutational profile of each cancer, we determined the relative burden of recurrent mutations to total mutations within each cohort, expressed as a recurrence score (RS). Recurrent mutations were defined as specific amino acid changes observed among greater than 1% of each cohort’s population. Mutational profiles, and thus RS, varied significantly between distinct cancers (Fig. S4). Several cohorts, notably adrenocortical carcinoma (ACC), acute myeloid leukemia (LAML), brain lower grade glioma (LGG), pheochromocytoma and paraganglioma (PCPG), thyroid carcinoma (THCA), thymoma (THYM), and uveal melanoma (UVM), revealed mutations occurring at high prevalence among the sequenced population. The recurrent mutations can be readily visualized as sharp peaks in the cancers’ mutational profiles. Such cohorts were found to have high recurrence scores (RS). Other cohorts, such as skin cutaneous melanoma (SKCM) and ovarian carcinoma (OV), displayed mutational profiles with fewer pronounced recurring mutations (sharp peaks). These cohorts carried a higher enrichment of non-recurrent mutations, and thus were found to have lower RS (Fig. 2).

We catalogued the specific tumor mutations observed among these cohorts displaying a highly-recurrent mutational landscape. In the adrenocortical carcinoma (ACC) cohort, 0.29% of all pooled missense mutations were in the *ZNF517* gene (p.V349A), and 0.29% of missense mutations were recurrent *GARS* (p.P42A) mutations. In uveal melanoma (UVM) cohort, 2.54% were recurrent *GNA11* (p.Q209P) mutations, 2.01% were recurrent *GNAQ* p.Q209P, and 0.75% *GNAQ* p.Q1209L. In thyroid carcinoma (THCA), 5.23% were *BRAF* p.V600E, 0.65% were *NRAS* p.Q61R, and 0.25% *HRAS* p.Q61R. In the acute myeloid leukemia (LAML) cohort, 1.38% of missense mutations were in *DNMT3A* gene (p.R882H), 1.05% were *IDH2* p.R140Q, and 0.79% were *IDH1* p.R132C. In pheochromocytoma and paraganglioma (PCPG), 0.64% of mutations were recurrent *HRAS* p.Q61R, and 0.36% were *CHEK2* p.K152E.

### Cancers with low recurrence scores (RS) show survival benefit from high TEMMB

**Figure 3:**
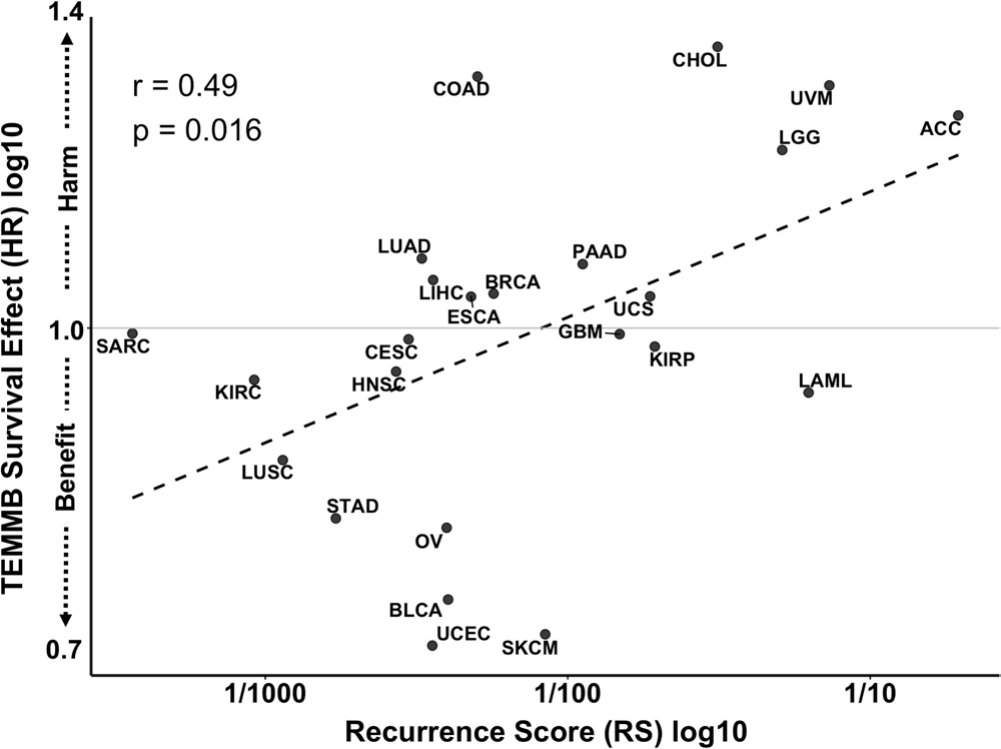
Correlation of log-adjusted mutational burden survival Hazard Ratios (HR) with cohorts’ log-adjusted recurrence scores (RS). Cancers with high recurrent mutation enrichment showed survival harm with increasing TEMMB, while tumors with low recurrent missense mutation burden tended to show survival benefit (r = 0.49, p = 0.016).

We identified a significant positive correlation (r = 0.49, p = 0.016) among all cancer cohorts between the survival effect, or Hazard Ratio (HR), of adjusted-TEMMB and cancer recurrence score (RS) (Fig. 3). Cancers with low RS tended to exhibit survival benefit (HR < 1) with increased adjusted-TEMMB. Conversely, cancers with high RS were observed to have a decrease in survival (HR > 1) with increased adjusted-TEMMB. Testing an alternate recurrence cutoff of 5% (traditional cutoff for minor allele frequency) confirmed a significant positive correlation: r = 0.66, p = 0.002 (Table S1).

## Discussion

Exonic missense mutation distribution displays considerable variability among cancers studied in TCGA. We identified cutaneous melanoma and lung squamous cell carcinoma as the tumors with the highest TEMMB, and acute myeloid leukemia and thyroid carcinoma as among the lowest. These results were consistent with previously-reported mutational burden distribution^12^. Somatic missense mutations strongly contribute to the generation of novel tumor epitopes. Understanding whether a more highly-immunogenic tumor carries a direct link to mutational burden could provide a mechanistic explanation for observed clinical survival patterns. In our results, TEMMB was closely correlated with TEMB among all TCGA cohorts, supporting TEMMB’s role as a robust proxy for TEMB.

Exonic missense mutational burden showed strong consistent positive association with age, supporting current understanding of human mutagenesis. While age-related mutagenesis rates do vary between individuals and tissue types, a consistent positive correlation between mutational load and age has been supported by animal and human research^13–18^. Several “clock-like” mutational signatures may be contributory to this chronological mutagenesis phenomenon^19^.

Interestingly, low tumor stage was correlated (after Bonferroni adjustment) with high TEMMB in breast carcinoma, colon and rectal adenocarcinoma, and uveal melanoma. Chromosomal and microsatellite instability (MSI) are observed in early stages of adenomas, and significant chromosomal instability has been proposed as an underlying feature present prior to malignant transformation^20–22^. Low-stage adenocarcinoma tumors may thus carry higher mutational loads due to the pronounced underlying genomic instability. Although the role of immune therapy is not yet strongly established in colorectal cancer (CRC), the immune tumor microenvironment in CRC is an important factor in disease progression^23,24^. Likewise, breast carcinogenesis has been proposed to be regulated by innate and adaptive inflammatory responses^25^. It is possible that during progression towards high-stage adenocarcinoma tumors in breast and colorectal cancers, highly-immunogenic or high-TEMMB cells are cleared through immune targeting and elimination, thus selecting for a population of low-TEMMB cells with low neoantigen loads. Uveal melanoma has a low mutational burden which has been suggested as a possible reason for low success of immunotherapy in its treatment^26^. Given the high propensity for rapid metastasis in uveal melanoma, it is possible that intercepting such tumors at an early stage may partially be explained by a higher mutational load and thus more favorable immune response.

Driver mutations impart tumor growth advantage and are positively selected in cancer evolution, while biologically inert passengers accumulate without directional selection over the tumor growth timespan^27^. Many established bioinformatics methods to study drivers rely on techniques that identify recurrent mutations^28^, and thus we quantified recurrent and non-recurrent mutations to serve as proxy for relative amounts of drivers and passengers within a cancer type. Our results suggest high TEMMB tends to confer survival benefit in cancers with more non-recurrent (likely passenger) mutations, and decreased survival in cancers with high recurrent (likely driver) fractions. We propose that in malignancies with large enrichments of non-recurrent mutations, high TEMMB marks a high passenger count, and increasing passenger mutation load increases neoantigen presentation^29^ without imparting additional growth advantage or aggressiveness. Our observed benefit with high TEMMB supports literature findings for melanoma^6^ and ovarian carcinoma^2^. In cases of malignancies with higher relative amounts of recurrent or driver mutations, for instance in adrenocortical carcinoma (ACC), uveal melanoma (UVM), and brain lower grade glioma (LGG), high mutational burden correlates with increased drivers of aggressiveness and invasion. In our study, increasing TEMMB showed a trend towards survival harm in these highly somatically-recurrent tumors.

Recent work has suggested a “double-edged” effect of increased DNA variants, noting that on the one hand, high DNA variation increases accumulation of drivers which are beneficial to tumor adaptation; conversely, high concurrent passenger loads may outweigh the driver effects^30^. Our results suggest a model for improved understanding of the variable manifestations of this molecular tug-of-war among a variety of cancer types. We found the underlying mutational landscape of DNA changes to be quite variable among malignancies documented in TCGA. A group of cancers such as adrenocortical carcinoma, uveal melanoma, and brain glioma emerged as a “driver-enriched” class, while a second group – including cutaneous melanoma and ovarian carcinoma – emerged as a “passenger-enriched” class. Increasing DNA variation in these two classes, quantified as TEMMB, yielded opposing survival effects. Our findings highlight TEMMB as an independent survival biomarker with potential utility for risk-stratification and identification of those patients who may benefit from immunotherapy. Classification of malignancies into driver- or passenger-rich classes may also aid in identifying suitable candidate cancers for immune therapy trials.

The study was limited by the following factors: first, TCGA describes exome sequences, and thus mutations in noncoding regions could not be analyzed. Thus, TEMMB reflects specifically the exonic mutational burden rather the full genome scale. It is possible that non-coding DNA contributes significantly to survival, and further study with comprehensive full genome sequencing may help elucidate such effects. Second, details of therapy and treatment course were available not for all patients, and thus we were unable to systematically study effect modification and confounding by treatment differences. Third, *in-silico* findings are important for discovery of novel relationships and insights in tumor biology, but *in-vivo* studies are required to further analyze mechanisms by which TEMMB affects tumor immune surveillance, metabolic, and growth properties. Future work will focus on analysis of immunological mechanisms responsible for clearing high-TEMMB tumors with a low enrichment of recurrent mutations. Lastly, the study is also significantly limited by a lack of controlled population-based recruitment among the TCGA cohorts. We adjusted TEMMB to account for recruitment center to partially address this limitation. However, future work would benefit from a study with more clearly and regularly ascertained cohorts.

Our overall analyses suggest that positive and negative TEMMB effects on survival may depend on the enrichment of underlying recurrent mutations. Cancers with higher proportions of non-recurrent and thus likely passenger mutations showed survival benefit with high TEMMB, while cancers with higher recurrent mutation fractions (likely drivers) revealed a decrease in survival. Mutational signatures for some cancers might contribute significantly to overall TEMMB (e.g. UV-signature in the cutaneous melanoma cohort), thus, in part environmental effects contribute to the TEMMB survival effect. These findings highlight the relationship of tumor mutational burden to driver and passenger effects. Understanding how tumor mutational burden correlates with clinical outcomes for certain classes of malignancies will help guide clinical decisions regarding TEMMB as a useful biomarker for predicting survival and response to immunotherapy.

## Methods

R statistical language (Version 3.4.4)^31^ with ‘RTCGAToolbox’^32^, ‘MASS’^33^, ‘survminer’^34^, ‘forestplot’^35^ were used for analysis and plotting. We obtained somatic mutation and clinical data for 31 cancer cohorts in The Cancer Genome Atlas (TCGA). 6947 individuals had available mutation data; 6717 of the set had complete clinical data on age, sex, and stage; 2113 patients were deceased and had available time-to-death survival data.

We examined individuals with maximum TEMMB value in each cohort, excluding those with TEMMB greater than triple of the next largest TEMMB value. As an initial quality control (QC) step, 10 (0.1% of total) samples were excluded as outliers potentially representing technical batch effects in tumor DNA analysis. Pearson’s correlation was used to examine the relationship between TEMMB and TEMB across all cohorts. We then analyzed the relationship between TEMMB and patients’ clinical factors. Negative binomial regression was used to model TEMMB as a function of age (continuous variable: “years”), sex (categorical variable: “male” and “female”), tumor stage (categorical variable: “low” defined as Stage 0, I, II, “high” defined as Stage III, IV), and recruitment center (categorical variable). Sex was omitted from the model for those cancers affecting exclusively one gender – cervical squamous cell carcinoma (CESC), ovarian carcinoma (OV), prostate adenocarcinoma (PRAD), uterine corpus endometrial carcinoma (UCEC), and uterine carcinosarcoma (UCS). Staging data was not available for glioblastoma multiforme (GMB), acute myeloid leukemia (LAML), brain lower grade glioma (LGG), pheochromocytoma and paraganglioma (PCPG), prostate adenocarcinoma (PRAD), sarcoma (SARC), and thymoma (THYM).

Next, we examined the effect of TEMMB on survival. We considered the residuals obtained from the negative binomial regression models as the effective TEMMB adjusted for age, sex, stage, and recruitment center. We used these residuals as inputs to Cox-proportional hazards models to predict survival (in days) as a function of effective TEMMB. Survival effects were expressed as hazard ratios (HR), which can be defined as the effective hazard per day conferred by effective TEMMB. Because TEMMB is an overdispersed count variable, it was adjusted well through negative binomial regression. The significance of Cox-proportional hazards models was calculated with two-sided Wald tests. Survival analysis for all 31 cohorts is reported in Figure S5A. We observed that in certain cohorts, such as pheochromocytoma and paraganglioma (PCPG), fewer than 10 patients were tracked until death, with the majority lost to follow-up. In such cases, we suspected that the survival analysis was dominated by censored data points (Fig. S5B). Thus, we performed an additional QC step by excluding cohorts in the bottom 5^th^, 10^th^, and 20^th^ percentiles of number of non-censored events. Results upon stringent exclusion of the bottom 20^th^ percentile of cohorts are reported in the main text.

We aggregated all nonsynonymous missense mutations among all individuals in each cancer. Missense variants resulting in identical amino acid changes were aggregated as one specific variant type. Recurrent mutations were defined as those variants exceeded 1% prevalence in the cohort, which is the traditional allele frequency cutoff for eliminating rare DNA variation^36,37^. A somatic recurrence score (RS) was calculated as the fraction of total mutations in the cohort’s pool comprised by recurrent mutations as defined above:

$${RS}=\frac{\sum {Recurrent}\,{Missense}\,{Variants}}{\sum {All}\,{Missense}\,{Variants}}$$

A RS was assigned to each cancer type, and the correlation between log_10_-adjusted survival HR and log_10_-adjusted RS was computed with Pearson’s correlation. To demonstrate robustness to parameter choice, an additional recurrent mutation prevalence definition of 5% (traditional Minor Allele Frequency cutoff for common DNA variation^38^) was tested.

## Supplementary information

Supplementary Information
