# Supplementary material for: Burden of unique and low prevalence somatic mutations correlates with cancer survival
Source: Sci Rep. 2019 Mar 19;9:4848. doi: 10.1038/s41598-019-41015-5 (PMC6425006; doi:10.1038/s41598-019-41015-5)
Supplement: Supplementary file 1 — Supplementary Information [file 41598_2019_41015_MOESM1_ESM.pdf]

Supplementary information

Burden of unique and low prevalence somatic mutations correlates with cancer survival

Klebanov, N[1]; Artomov, M[2,3]; Goggins, WB[4]; Daly, E[2]; Daly, MJ[2,3]; Tsao, H[1*].

Addresses
[1]Wellman Center for Photomedicine, Massachusetts General Hospital, Harvard Medical School, Boston, MA, USA
[2]Analytic and Translational Genetic Unit, Massachusetts General Hospital, Boston, MA, USA
[3]Broad Institute, Cambridge, MA, USA
[4]School of Public Health and Primary Care, The Chinese University of Hong Kong, Hong Kong
*Corresponding author: htsao@mgh.harvard.edu

Summary
Figure S1. Distribution of tumor exonic missense mutational burden (TEMMB) among 31 cancer cohorts in TCGA.

Figure S2. Outliers in exonic missense mutations in red. Outliers were defined as individuals with the maximum observed TEMMB who had a TEMMB value greater than triple of the next largest TEMMB value. Overall, 10 (0.1% of total) samples were excluded as outliers.

Figure S3. Correlations of tumor exonic missense mutation burden (TEMMB) to total exonic mutational burden (TEMB) for each TCGA cancer cohort. Pearson's r ranges from 0.88 to 1.

Figure S4. Representation of individual missense mutations in the cohort population. High burden of recurrent mutations is observed among ACC, LAML, LGG, PCPG, THCA, THYM, and UVM. There are no apparent individual recurrent mutations prevalent among the remaining cancers.

Figure S5. A. TEMMB survival effects for all cohorts regardless of number of non-censored events. B. Kaplan-Meier curves for pheochromocytoma and paraganglioma cancer (PCPG). Note the low number of non-censored data points resulting in biased statistical significance.

Table S1. To test the robustness of the observed correlation, we tested excluding cohorts at/below the 5th, 10th, and 20th percentiles, and tested two different thresholds of 1% and 5% for defining mutation recurrence. The correlation was positive and statistically significant at all tested conditions.

Supplementary Figure 1

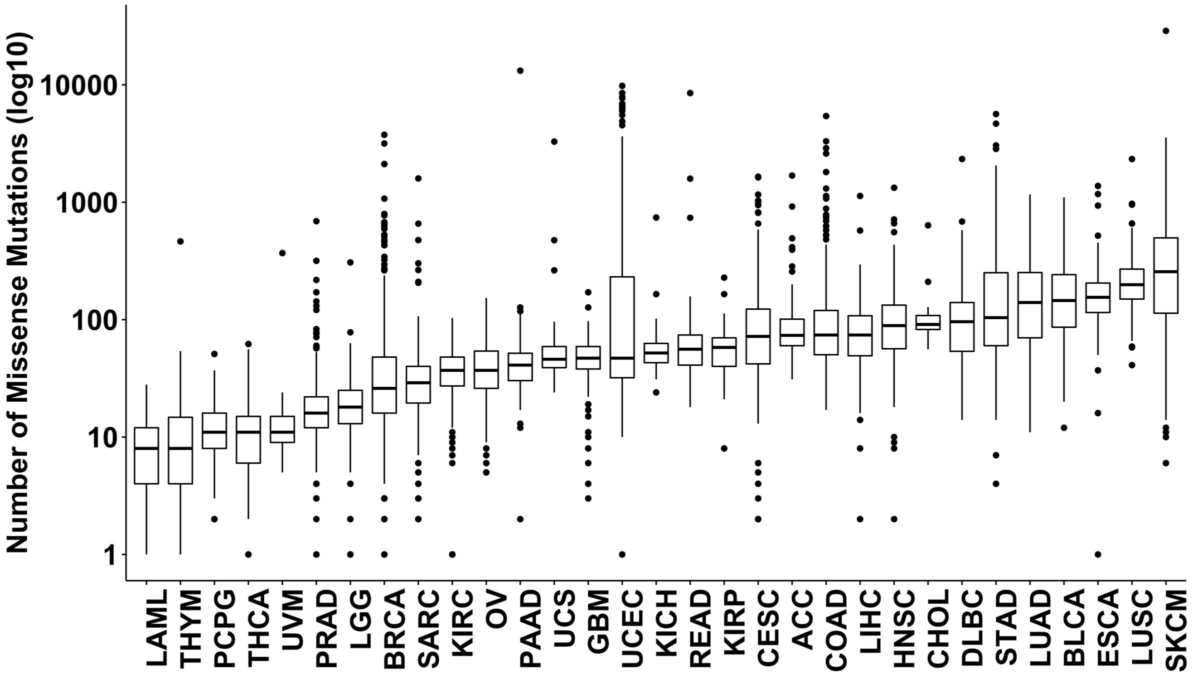

Supplementary Figure 1. Distribution of tumor exonic missense mutational burden (TEMMB) among 31 cancer cohorts in TCGA.

Supplementary Figure 2

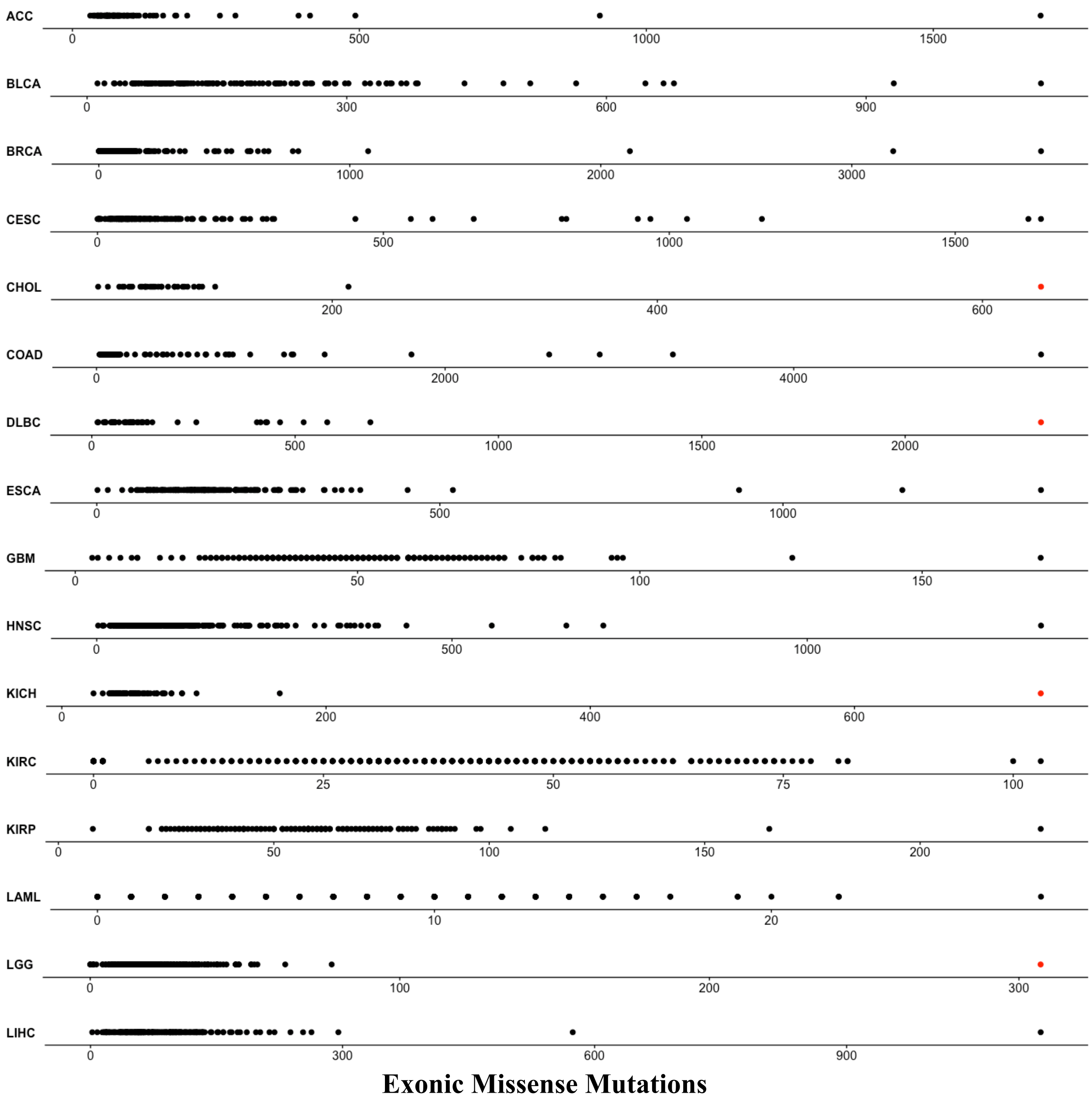

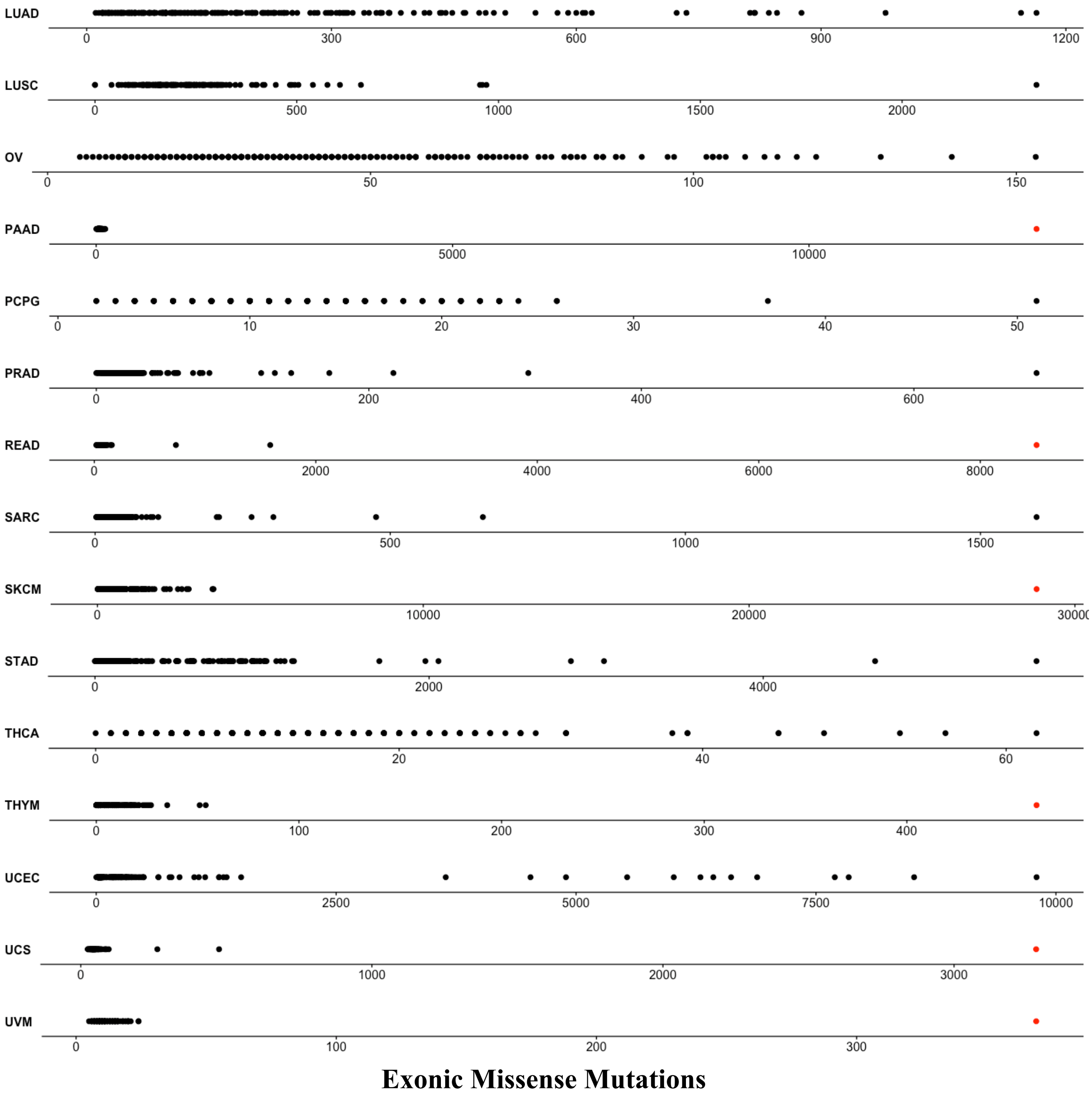

Supplementary Figure 2. Outliers in exonic missense mutations in red. Outliers were defined as individuals with the maximum observed TEMMB who had a TEMMB value greater than triple of the next largest TEMMB value. Overall, 10 (0.1% of total) samples were excluded as outliers.

Supplementary Figure 3

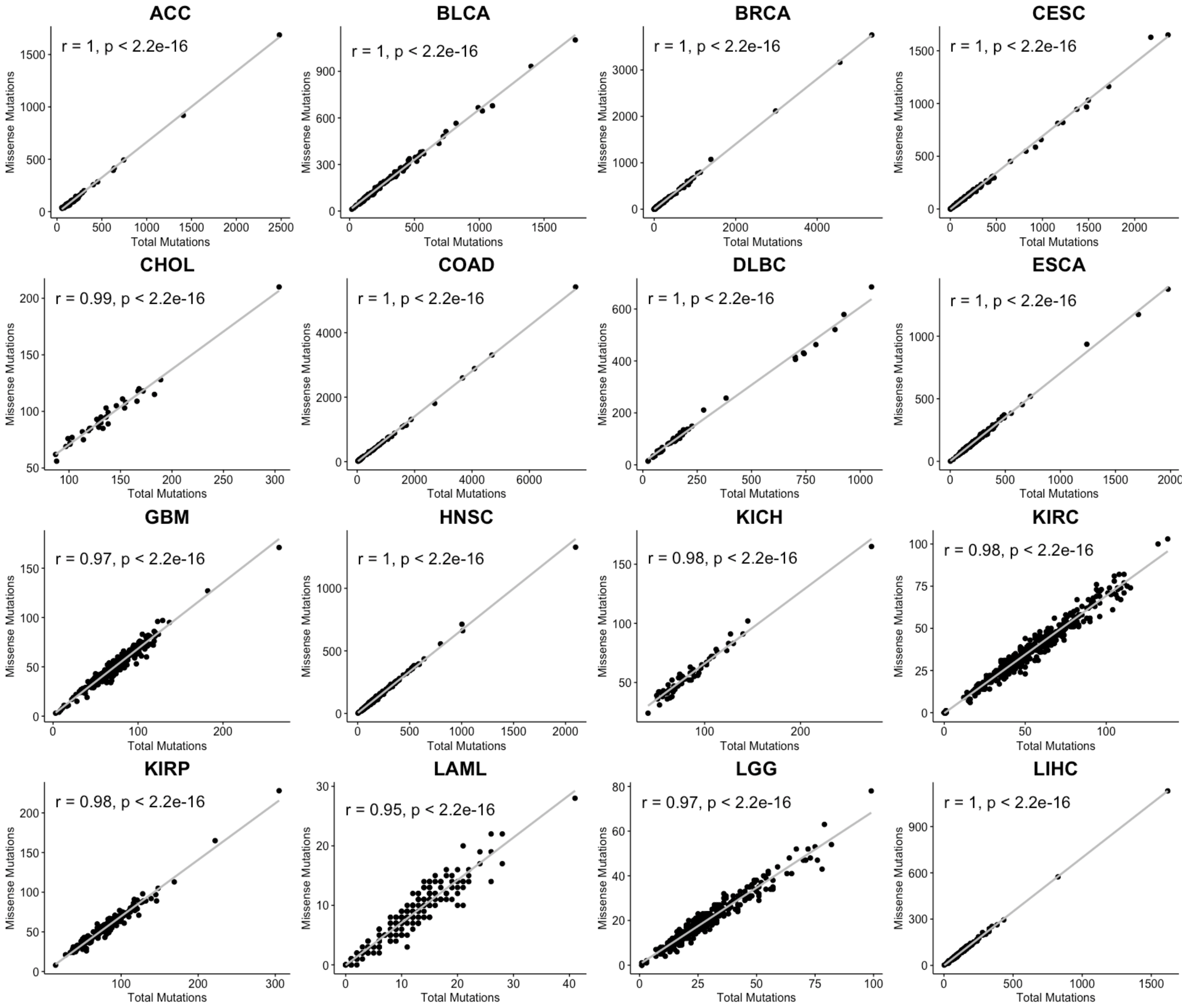

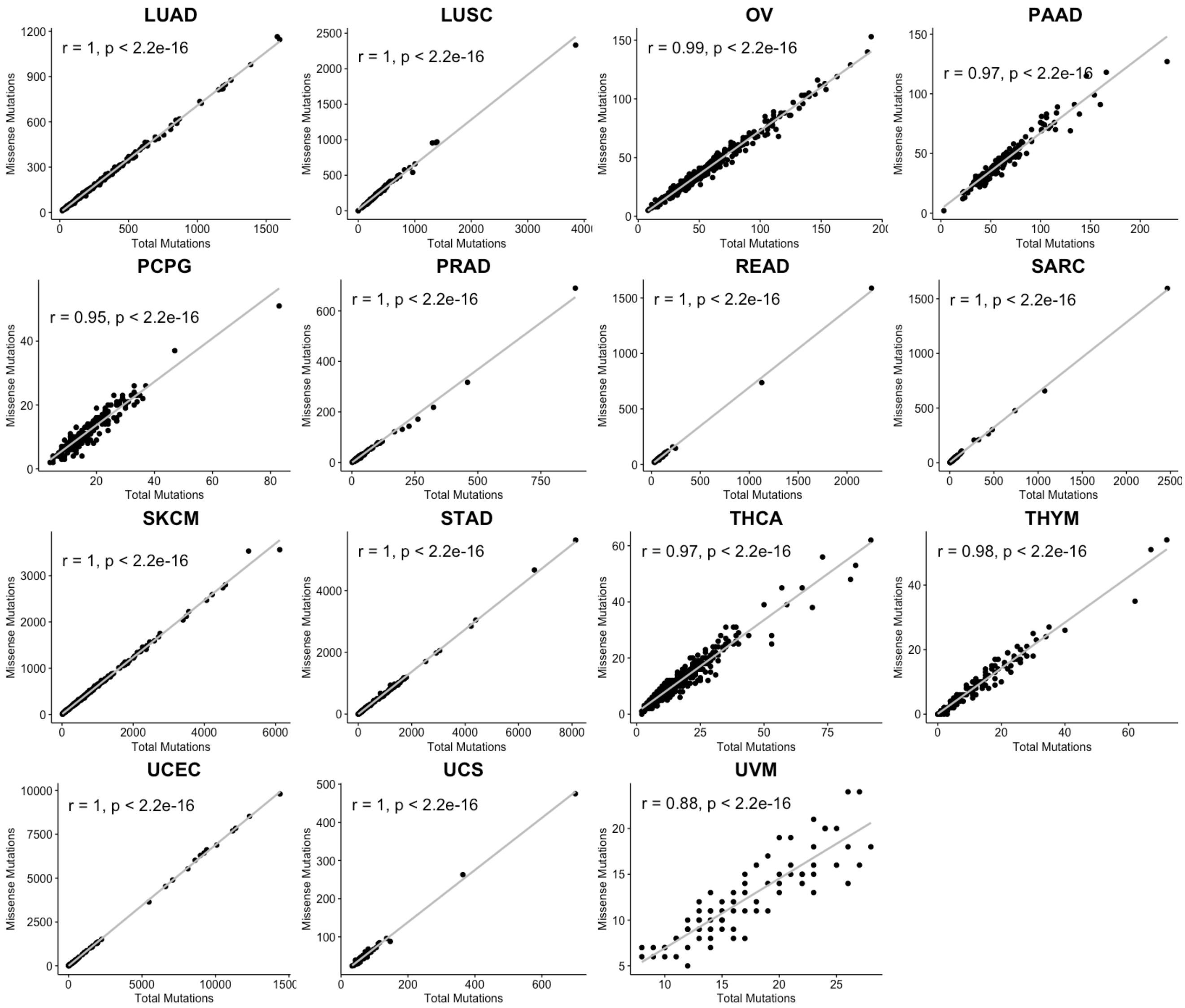

Supplementary Figure 3. Correlations of tumor exonic missense mutation burden (TEMMB) to total exonic mutational burden (TEMB) for each TCGA cancer cohort. Pearson's r ranges from 0.88 to 1.

Supplementary Figure 4

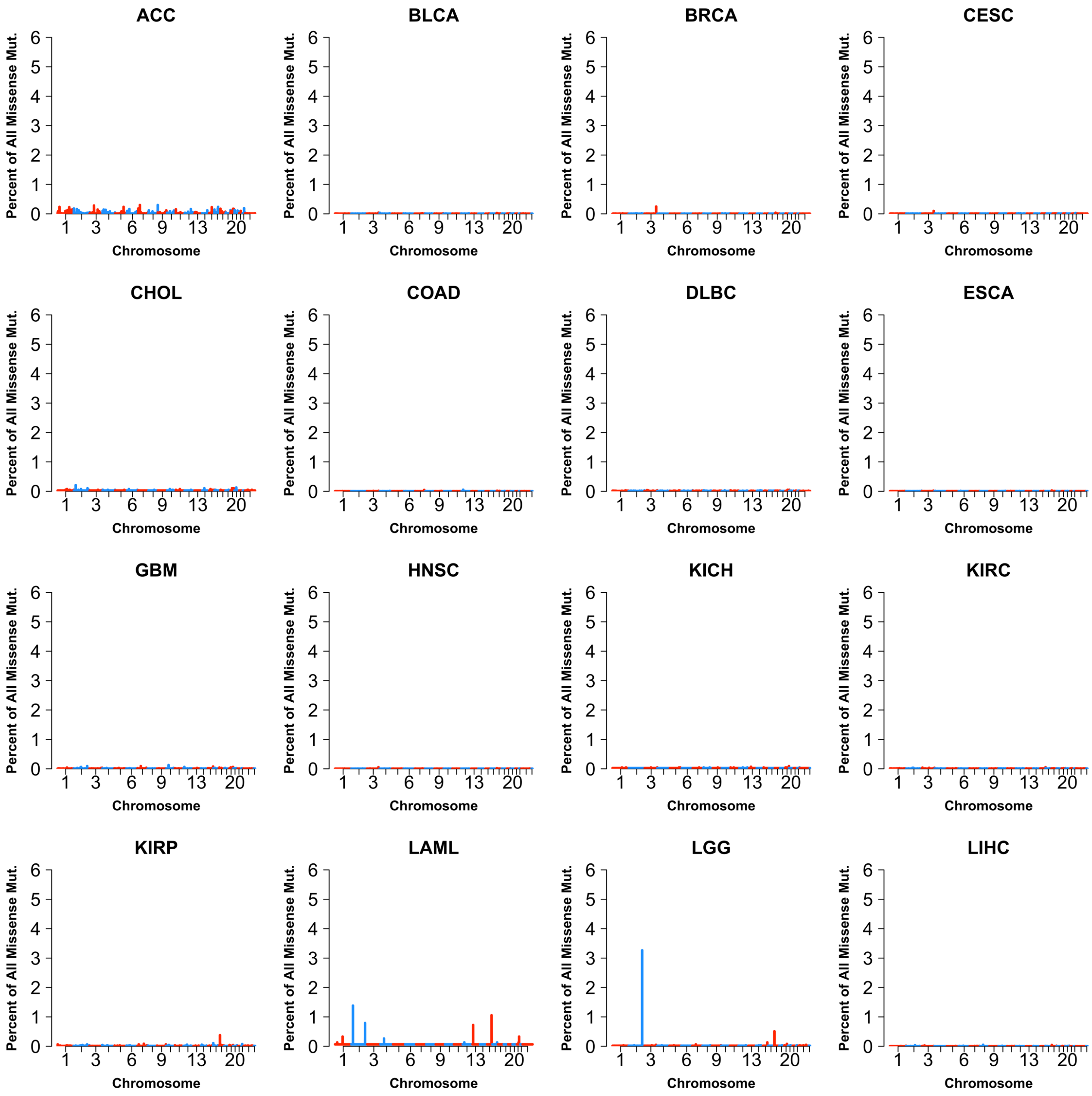

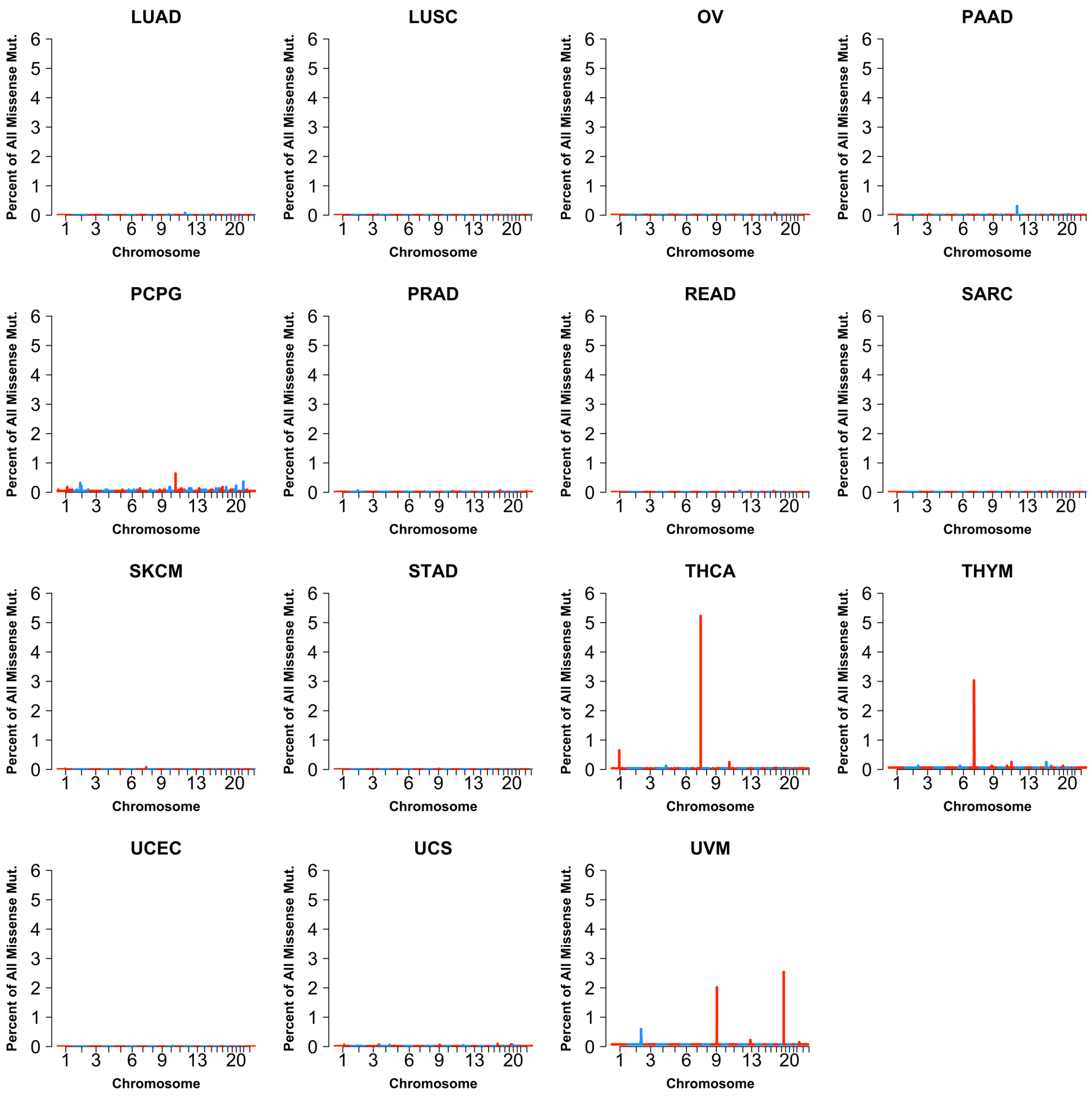

Supplementary Figure 4. Cumulative missense mutations (with mutations aggregated as independent variants if they produced identical amino acid changes) as a percent of all missense mutations in the cohort pool. Pronounced peaks corresponding to mutations frequently recurring within a particular cohort population are observed in ACC, LAML, LGG, PCPG, THCA, THYM, and UVM. The remaining cohorts display flatter mutational profiles with fewer pronounced recurring mutations.

Supplementary Figure 5

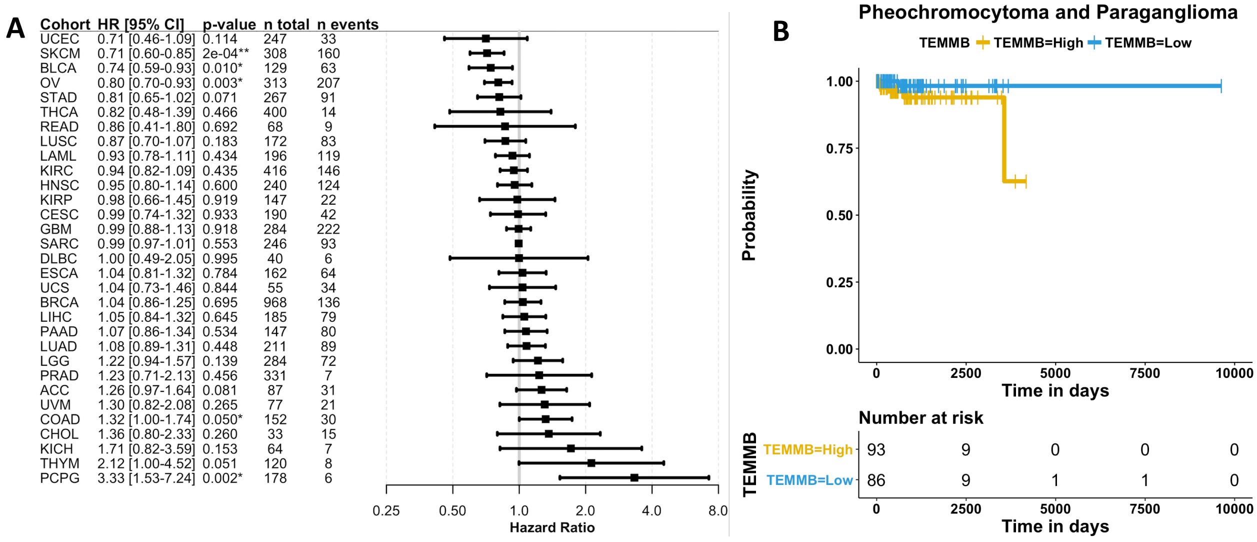

Supplementary Figure 5. A. TEMMB survival effects for all cohorts regardless of number of non-censored events. B. Kaplan-Meier curves for pheochromocytoma and paraganglioma cancer (PCPG). Note the low number of non-censored data points resulting in biased statistical significance.

SUPPLEMENTARY TABLES

Supplementary Table 1

		Pearson's r			p value	
		Recurrence Definition			Recurrence Definition	
		1%	5%		1%	5%
Cohort Percentile Excluded by Number of Noncensored Events	0th	0.42	0.43	0th	0.017	0.036
	5th	0.39	0.55	5th	0.037	0.008
	10th	0.42	0.55	10th	0.029	0.008
	20th	0.49	0.66	20th	0.016	0.002
